# Recurrence eigenvalues of movements from brain signals

**DOI:** 10.1186/s40708-021-00143-3

**Published:** 2021-10-15

**Authors:** Tuan D. Pham

**Affiliations:** Center for Artificial Intelligence, Prince Mohammad Bin Fahd University, Khobar, Saudi Arabia

**Keywords:** Eigenworms, Phenotypes, Behavioral genetics, Nervous system, Neurodegenerative diseases, Gait dynamics, Time series, Fuzzy recurrence eigenvalues, Nonlinear dynamics, Convolution

## Abstract

The ability to characterize muscle activities or skilled movements controlled by signals from neurons in the motor cortex of the brain has many useful implications, ranging from biomedical perspectives to brain–computer interfaces. This paper presents the method of recurrence eigenvalues for differentiating moving patterns in non-mammalian and human models. The non-mammalian models of *Caenorhabditis elegans* have been studied for gaining insights into behavioral genetics and discovery of human disease genes. Systematic probing of the movement of these worms is known to be useful for these purposes. Study of dynamics of normal and mutant worms is important in behavioral genetic and neuroscience. However, methods for quantifying complexity of worm movement using time series are still not well explored. Neurodegenerative diseases adversely affect gait and mobility. There is a need to accurately quantify gait dynamics of these diseases and differentiate them from the healthy control to better understand their pathophysiology that may lead to more effective therapeutic interventions. This paper attempts to explore the potential application of the method for determining the largest eigenvalues of convolutional fuzzy recurrence plots of time series for measuring the complexity of moving patterns of *Caenorhabditis elegans* and neurodegenerative disease subjects. Results obtained from analyses demonstrate that the largest recurrence eigenvalues can differentiate phenotypes of behavioral dynamics between wild type and mutant strains of *Caenorhabditis elegans*; and walking patterns among healthy control subjects and patients with Parkinson’s disease, Huntington’s disease, or amyotrophic lateral sclerosis.

## Introduction

*Caenorhabditis elegans* is a nematode or roundworm about 1 mm in length [1] and commonly used as a model organism in the study of genetics because of its powerful genetics and fully characterized simple nervous system [2, 3]. The nervous system of the *C. elegans* hermaphrodite consists of 302 neurons that form 118 morphologically distinct neuron classes [4]. These neurons activate many distinct stimulus modalities and then combine them to produce distinct patterns of behavior [5, 6].

The study of the movement of *C. elegans* is important because the knowledge gained from understanding the mechanism underlying the moving of these worms can be useful for discovering new characteristics in behavioral genetics. It is known that behavior is a visual display of sensitive and integrative information of nervous system function and plays as an effective measure for evaluating the effects of mutation or efficacy of drug treatment for animals [7]. Ultimately, such knowledge is expected to provide potential alternatives for better diagnosis and therapeutics of human disease [8].

An interesting study reported in [9] carried out an experiment on the movements of *C. elegans*. An explanatory summary of the study can be found online (https://ilovesymposia.com/2008/10/02/eigenworms/). In this study of the dimensionality and dynamics of *C. elegans*, the shape of a worm was modeled as a vector of 100 angles measured between 101 adjacent segments. Consequently, a 100 $\times$ 100 correlation matrix was then constructed and its 100 eigenvalues were computed. The study discovered that only 4 of the 100 eigenvalues accounted for more than 90% of the variability of worm shapes. These 4 eigenvectors define 4 fundamental worm shapes, which are called eigenworms. As a finding, the worm motion could be categorized into four characteristic shapes whose combination can describe any shape that the worm can form. The first two eigenworms, which correlate with a wave propagating along the body of a worm, contribute to the forward motion. The third eigenworm correlates with the curvature whose variations navigate the movement of a worm along its trajectory. Finally, the fourth eigenworm correlates with the movement of the worm’s head as it searches for food and traverses.

As an important step toward the elucidation of behavior in *C. elegans*, an ability to measure the difference between patterns of motion of these worm types is necessary to allow for pattern prediction that can provide insights into the control of the dynamics of movement [10]. It is therefore of interest to explore methods for quantifying and differentiating complexity in time and vision series of eigenworm motions between wild type and mutant strains of *C. elegans* [10, 11].

Neurodegenerative diseases affect many physical activities of the patients, particularly balance and movement. Many of these diseases are thought to be gene inheritance disorders, but the cause is largely unknown [12]. Diseases of degenerative nerves can be life-threatening and currently have no cure. Therefore, effective treatments, including medications and surgery, may help the patients improve mobility and relieve pain. Gait impairment is a common symptom in neurodegenerative disorders. Specifically, gait variability, which is the stride-to-stride fluctuations measured with time, has been known to be associated with neurodegeneration [13].

Several studies on gait dynamics in human neurodegeneration have been reported in literature [14–19]. These studies attempted to discover new features of gait time series mainly used for pattern classification of healthy control (HC), Parkinson’s disease (PD), Huntington’s disease (HD), and amyotrophic lateral sclerosis (ALS) subjects. However, quantitative characterization of gait and its impairments has not been well investigated. New computational methods that can provide scalar values to represent some attributes of the gait dynamics of the HC and diseases can be useful because these quantitative descriptors can used for early disease detection or physiological makers.

In this paper, the method for computing the largest eigenvalue of a convolutional fuzzy recurrence plot of time series [20] is investigated for measuring the quantity of the complexity of behavioral and moving patterns of eigenworm behavioral phenotypes of different *C. elegans* strains, and patients with neurodegenerative disorders (PD, HD, and ALS) and HC subjects. Here, based on the concepts of nonlinear dynamics and chaos theory, time series of the time series are reconstructed into phase space sets and their matrices of spatial recurrence features extracted. A convolutional kernel is then iteratively applied on these recurrence features to encode feature invariance. Finally, the largest eigenvalues of the deepest convolutional recurrence matrices are determined and used as indicators of complexity of motion or walking patterns in different worm types or human subjects, respectively.

## Materials and methods

### Eigenworm data

The time-series dataset used in this study was described in [21]. The data relate to 258 traces of worms converted into eigenworm time series. Worm motions on an agar plate were recorded and a range of human-defined features measured [22]. The worm outline was extracted, where each frame of the worm motion was captured and represented with amplitudes when the shape was projected onto the eigenworms. There are five classes of *C. elegans*: N2, goa-1, unc-1, unc-38, and un63. The N2 is wild type (normal), and the other four are mutant strains. This dataset, which is publicly available online [23], consists of the time series of the first dimension or first eigenworm. Table 1 shows the number and length of the time series of each of the five worm types.

**Table 1:** Caenorhabditis elegans data

Worm class	Number of time series	Length
N2 (wild type)	109	900
goa-1 (mutant)	44	900
unc-1 (mutant)	35	900
unc-38 (mutant)	45	900
unc-63 (mutant)	25	900

### Gait in neurodegenerative disease data

This third-party public database [24] includes gait signals recorded from patients with PD, HD, ALS, and HC subjects. The database also provides clinical information for each subject, including age, gender, height, weight, walking speed, and a measure of disease severity or duration. The signals were obtained using force-sensitive resistors, with output proportional to the force under the foot. Stride-to-stride measures of footfall contact times were derived from these signals. Detailed information about these data was provided in [25, 26]. Both time series of the left swing interval (LSI) and right swing interval (RSI) are used in this study. Both LSI and RSI were measured in seconds. Because these original time series have not been filtered, they are filtered using 1-D median filtering that applies a third-order one-dimensional median filter to the input time series and considers the signal to be zero beyond the endpoints. To ensure the time series of all subjects are of the same length, all the time series have a truncated length of the first 120 time points, which is equivalent to the original shortest time-series length. Table 2 shows the numbers of HC, PD, HD, and ALS subjects and truncated length of the LSI and RSI time series.

**Table 2:** Gait in neurodegenerative disease data

Cohort	Number of subjects	Length of LSI and RSI
HC	16	120
PD	15	120
HD	20	120
ALS	13	120

### Eigenvalues of recurrence dynamics

A sequence of values in time can be transformed into an object in space, which is called the phase space of a dynamical system, and the object in the phase space is referred to as the phase space set. Such a transformation is particularly useful because some properties underlying complex data can be more easily discovered from the phase space set than from the original time series [27]. According to the embedding theorem [28, 29], it is always possible to reconstruct the phase space from a time series. A popular method for the phase-space reconstruction is the technique of time delay [28, 29], which is applied for reconstructing the phase space of the eigenworm time series as follows.

Let ${\mathbf{t}} = (t_1, t_2, \dots , t_N)$ be a time series of scalar values generated by the eigenworm motion. Given *m* as an embedding dimension and $\tau$ as a time delay, the time series ${\mathbf{t}}$ can be reconstructed as ${\mathbf{X}} = ({\mathbf{x}}_1, {\mathbf{x}}_2, \dots , {\mathbf{x}}_M)^\text{{T}}$, where $M = N-(m-1) \tau$, and ${\mathbf{x}}_i = (t_i, t_{i+\tau }, t_{i+2\tau }, \dots , t_{i+(m-1)\tau })$. Now it can be seen that the time series ${\mathbf{t}}$ can be transformed into ${\mathbf{X}}$ that is expressed as a matrix, where each row is a phase-space vector ${\mathbf{x}}_i$, $i = 1, 2, \dots , M$.

Based on the phase space set reconstructed from a time series, a method for studying behaviors of systems of nonlinear dynamics is the recurrence plotting that examines the revisit or recurrence of the trajectory in the phase space [30]. A recurrence plot (RP) is a binary symmetrical matrix showing either pairs of time points at which the trajectory is at the same place (representing with a black dot) or not (representing with a white dot). As an extended algorithm of an RP, the construction of a fuzzy recurrence plot (FRP) [31] was introduced to overcome the difficulty for determining the threshold for similarity and limited binary expression of recurrence imposed by the RP method. An FRP represents the recurrence of the trajectory in the phase space as a grayscale image taking values in [0, 1], where 0 is a black pixel, 1 a white pixel, and other values gray pixels. The construction of an FRP works by partitioning ${\mathbf{X}}$ into a number of clusters, denoted as *c*, using the fuzzy *c*-means (FCM) algorithm [32]. The FCM assigns a fuzzy membership grade, denoted as $\mu _{ij}$ taking values in [0, 1], to each ${\mathbf{x}}_i$, $i=1, 2, \dots , M$, with respect to each cluster center ${\mathbf{v}}_j$, $j = 1, 2, \dots , c$. The fuzzy membership variable $\mu _{ij}$ expresses the degree that ${\mathbf{x}}_i$ possibly belongs to ${\mathbf{v}}_j$.

The FRP method applies the two following properties:

- Reflexivity:
  1 $$\begin{aligned} \mu _{ii} = 1, \, i=1, 2, \dots , M. \end{aligned}$$
- Symmetry:
  2 $$\begin{aligned} \mu _{ij} = \mu _{ji}, i = 1, \dots , M, j = 1, 2, \dots , c. \end{aligned}$$

The fuzzy relation between the pairs of the phase-space vectors ${\mathbf{x}}_i$ and ${\mathbf{x}}_k$; $i, k = 1, 2, \dots , M$ can be inferred by transitivity using the max–min composition operator [33] as

- Transitivity:
  3 $$\begin{aligned} \mu _{ik} = \max [\min (\mu _{ij},\mu _{jk})], j = 1, \dots , c. \end{aligned}$$

As a result, an FRP is a square grayscale image or matrix defined as

4 $$\begin{aligned} \text {FRP} = \begin{bmatrix} \mu _{11} &{} \mu _{12} &{} \dots &{} \mu _{1M}\\ \mu _{21} &{} \mu _{22} &{} \dots &{} \mu _{2M}\\ \dot{.} &{} \dot{.} &{} \dot{.} &{} \dot{.} \\ \mu _{M1} &{} \mu _{M2} &{} \dots &{} \mu _{MM} \end{bmatrix}. \end{aligned}$$

By using on the concept of convolutional filtering in deep learning for invariant feature extraction of image objects, where convolutional layers are building blocks for the design of convolutional neural networks [34], a deep convolutional FRP, denoted as cFRP, can be performed by a series of convolutions of the FRP and a convolution kernel as [20]

5 $$\begin{aligned} \text {cFRP} = w \circledast \text {FRP}, \end{aligned}$$

where *w* is a convolution kernel and $\circledast$ denotes the convolutional operator.

In this study, the kernel is used as a sharpening-effect $3 \times 3$ matrix, whose elements are given as

6 $$\begin{aligned} w = \begin{bmatrix} 0 &{} -1 &{} 0 \\ -1 &{} 5 &{} -1 \\ 0 &{} -1 &{} 0 \end{bmatrix}. \end{aligned}$$

After each step of the convolution, the rectified linear unit (ReLU) is then applied to eliminate negative values of the cFRP. The ReLU for a cFRP returns a zero or positive value to each element $\text {cFRP}(i,k)$ as

7 $$\begin{aligned} \text {cFRP}^*(i,k) = \max [0,\text {cFRP}(i,k)]; i, k = 1, 2, \dots , M. \end{aligned}$$

The next step is to reduce the size of the convolved FRP by using a pooling operator, which aims to reduce the dimensionality of a convolutional feature map but still keeps the useful information. Different types of pooling include the max, average, or sum operator. A popular choice for the pooling of a convolutional matrix in deep learning is the max pooling operator. Let a set of pooling regions be $\Omega = (\Omega _1, \Omega _2, \dots , \Omega _Q)$, where $\Omega _q = (\omega _{q,1}, \omega _{q,2}, \dots , \omega _{q, m \times m})$, $q = 1, 2, \dots , Q$. The number of pooling regions *Q* within a convolutional matrix is determined by the pool size $m \times m$ and stride that is the step size for traversing the convolutional FRP. The max pooling that operates on a pooling region of size $m \times m$, denoted as $P_{\max }$, is defined as

8 $$\begin{aligned} P_{\max } (\Omega _q) = \max _{1 \le l \le m \times m} (\omega _{q,l}). \end{aligned}$$

Finally, the eigenvalues of a cFRP having a certain small square matrix size can be determined, where the largest eigenvalue, denoted as $\lambda _{\max }$, is used as the characteristic value of recurrence dynamics.

*Procedure for computing the largest eigenvalue of a cFRP:*

1. Input: an $N \times N$ FRP
2. Given *w*, ReLU, pool size, and stride
3. Given *n* as the desired final $n \times n$ convolved FRP
4. While $$N > n$$
5. Perform convolution of the FRP and *w*.
6. Apply ReLU on the convolved FRP.
7. Perform max pooling on the convolved FRP.
8. End While loop.
9. If $N = n$, compute the largest eigenvalue of the $n \times n$ cFRP.

**Fig. 1:**
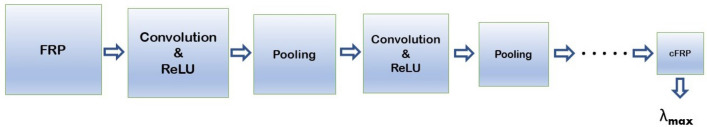
Process for computing the largest eigenvalue of a convolutional FRP

Figure 1 shows the iterative process for computing the final convolved FRP whose largest eigenvalue is determined.

## Results

### Eigenworm behavioral phenotypes

**Fig. 2:**
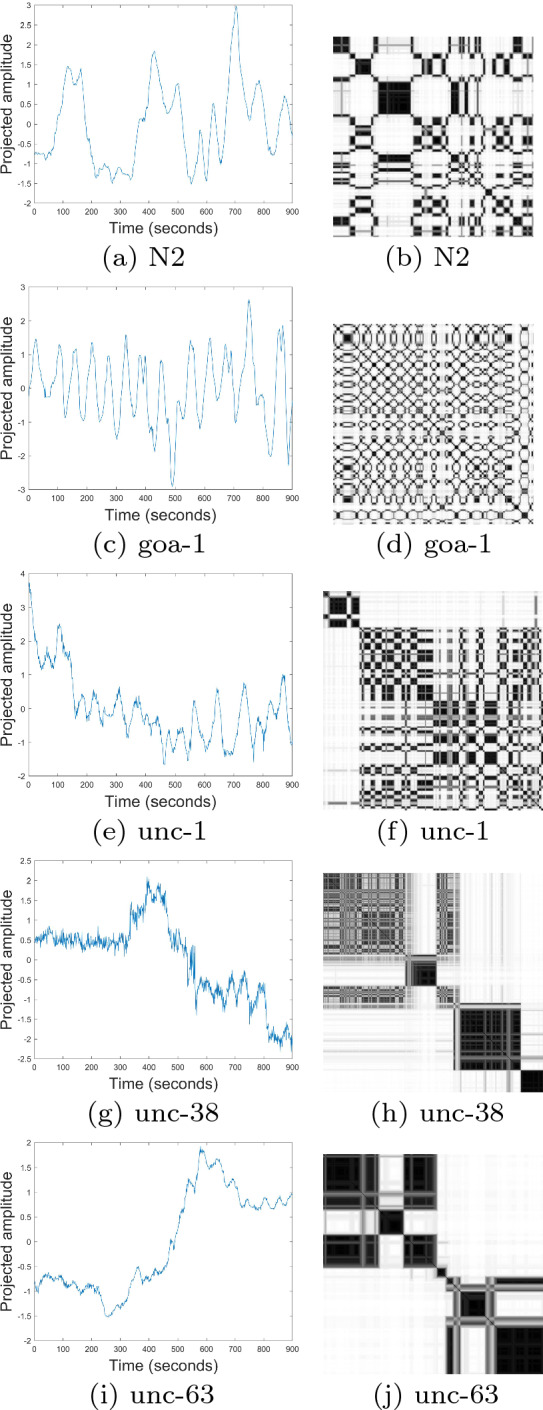
Time series (left) and fuzzy recurrence plots (right) of wild type (**a**, **b**) and mutant (**c**–**j**) *Caenorhabditis elegans*

To determine the largest eigenvalues from the time series of the *C. elegans* behavioral phenotypes presented in the foregoing section, the embedding dimension *m* = 4 was selected based on the identification of the four principal dimensions of the eigenworms, time delay $\tau$ = 1, and number of clusters *c* = 3, 5, and 7. Figure 2 shows some time series and FRPs of the five *C. elegans* classes, where the FRPs were constructed with *m* = 4, $\tau$ = 1, and *c* = 3. It can be seen that, the texture of the FRP of N2 (wild type or normal) is distinctive from the four mutant classes (goa-1, unc-1, unc-38, and unc-63), while the texture patterns of (goa-1 and unc-1) and (unc-38 and unc-63) are similar to each other.

To compute the largest eigenvalues, the size of the final cFRPs for the five *C. elegans* classes was set as *n* = 2, that is a $2 \times 2$ matrix, to allow the deepest feature extraction based on the deep-learning approach.

Table 3 shows the average largest eigenvalues and standard deviations (SDs) of the *C. elegans* behavioral dynamics with embedding dimension = 4 and different numbers of clusters. Table 3 also shows the *p*-values, 95% confidence intervals (95% CI), and 99% confidence intervals (99% CI) of largest eigenvalues.

**Table 3:** Average largest eigenvalues and confidence intervals of eigenworm recurrence behavior

Worm class	Mean ± SD	*p*-value	95% CI	99% CI
*m* = 4, $\tau$ = 1, *c* = 3				
N2 (wild type)	5.4800 ± 0.8322	5.5114e-91	(5.3220, 5.6380)	(5.2710, 5.6890)
goa-1 (mutant)	5.5729 ± 0.8274	1.1304e-37	(5.3213, 5.8244)	(5.2367, 5.9091)
unc-1 (mutant)	5.5454 ± 0.6354	6.8754e-34	(5.3272, 5.7637)	(5.2524, 5.8385)
unc-38 (mutant)	5.5037 ± 0.7124	4.4008e-41	(5.2896, 5.7177)	(5.2178, 5.7896)
unc-63 (mutant)	5.7802 ± 1.0175	5.5225e-20	(5.3602, 6.2002)	(5.2110, 6.3494)
*m* = 4, $\tau$ = 1, *c* = 5				
N2 (wild type)	5.2741 ± 0.4828	2.1497e-114	(5.1825, 5.3658)	(5.1529, 5.3954)
goa-1 (mutant)	5.4309 ± 0.5718	5.4091e-44	(5.2571, 5.6048)	(5.1986, 5.6633)
unc-1 (mutant)	5.4701 ± 0.7688	6.4414e-31	(5.2060, 5.7342)	(5.1155, 5.8247)
unc-38 (mutant)	5.3694 ± 0.5343	4.7729e-46	(5.2089, 5.5299)	(5.1550, 5.5838)
unc-63 (mutant)	5.6012 ± 0.8915	5.2205e-21	(5.2333, 5.9692)	(5.1026, 6.0999)
*m* = 4, $\tau$ = 1, *c* = 7				
N2 (wild type)	5.2862 ± 0.4946	2.2152e−113	(5.1923, 5.3801)	(5.1620, 5.4104)
goa-1 (mutant)	5.5029 ± 0.6823	5.5972e−41	(5.2955, 5.7104)	(5.2257, 5.7802)
unc-1 (mutant)	5.2543 ± 0.5388	1.6446e−35	(5.0692, 5.4394)	(5.0058, 5.5028)
unc-38 (mutant)	5.3543 ± 0.4108	5.5942e−51	(5.2308, 5.4777)	(5.1894, 5.5191)
unc-63 (mutant)	5.3377 ± 0.3878	4.3521e−29	(5.1777, 5.4978)	(5.1208, 5.5546)

To compare the results obtained from the largest eigenvalues with one of the most popular methods for quantifying the fluctuation or predictability of time series, the method of sample entropy (SampEn) [35] was used to compute the SampEn values of the time series of the five *C. elegans* types. Input parameters for computing SampEn values of the time series were selected as: embedding dimension *m* = 4, time delay $\tau$ = 1, and threshold $\delta$ = 0.1$\sigma$, 0.2$\sigma$, and 0.3$\sigma$, where $\sigma$ is the standard deviation of the time series (a practical and well-adopted selection of $\delta$ was suggested to be between 0.1$\sigma$ and 0.25$\sigma$ [36, 37]). Table 4 shows the average SampEn values and SDs of the *C. elegans* dynamics with different values for $\delta$ as well as the *p*-values, 95% CIs, and 99% CIs.

**Table 4:** Average SampEn values and confidence intervals of eigenworm recurrence behavior

Worm class	Mean ± SD	*p*-value	95% CI	99% CI
*m* = 4, $\tau$ = 1, $\delta$ = 0.1$\sigma$				
N2 (wild type)	0.3091 ± 0.2032	5.1478e−30	(0.2705, 0.3477)	(0.2580, 0.3601)
goa-1 (mutant)	0.4239 ± 0.1309	1.2866e−24	(0.3841, 0.4637)	(0.3707, 0.4770)
unc-1 (mutant)	0.4692 ± 0.2278	5.8924e−14	(0.3909, 0.5475)	(0.3641, 0.5743)
unc-38 (mutant)	0.2169 ± 0.1342	5.2455e−14	(0.1766, 0.2572)	(0.1630, 0.2708)
unc-63 (mutant)	0.1723 ± 0.1003	8.6758e−09	(0.1309, 0.2137)	(0.1162, 0.2284)
*m* = 4, $\tau$ = 1, $\delta$ = 0.2$\sigma$				
N2 (wild type)	0.1845 ± 0.1326	3.7199e−27	(0.1593, 0.2097)	(0.1512, 0.2178)
goa-1 (mutant)	0.2666 ± 0.0916	8.4893e−23	(0.2387, 0.2945)	(0.2294, 0.3038)
unc-1 (mutant)	0.2423 ± 0.1306	1.0195e−12	(0.1974, 0.2871)	(0.1820, 0.3025)
unc-38 (mutant)	0.1020 ± 0.0548	4.7314e−16	(0.0855, 0.1185)	(0.0800, 0.1240)
unc-63 (mutant)	0.0753 ± 0.0419	3.8214e−09	(0.0580, 0.0925)	(0.0518, 0.0987)
*m* = 4, $\tau$ = 1, $\delta$ = 0.3$\sigma$				
N2 (wild type)	0.1261 ± 0.0833	7.2039e−30	(0.1103, 0.1419)	(0.1052, 0.1470)
goa-1 (mutant)	0.2039 ± 0.0759	1.8169e−21	(0.1808, 0.2270)	(0.1731, 0.2347)
unc-1 (mutant)	0.1485 ± 0.0916	3.3498e−11	(0.1171, 0.1800)	(0.1063, 0.1908)
unc-38 (mutant)	0.0612 ± 0.0281	1.8702e−18	(0.0527, 0.0696)	(0.0499, 0.0725)
unc-63 (mutant)	0.0431 ± 0.0233	2.1791e−09	(0.0335, 0.0527)	(0.0301, 0.0562)

### Gait in neurodegenerative diseases

**Fig. 3:**
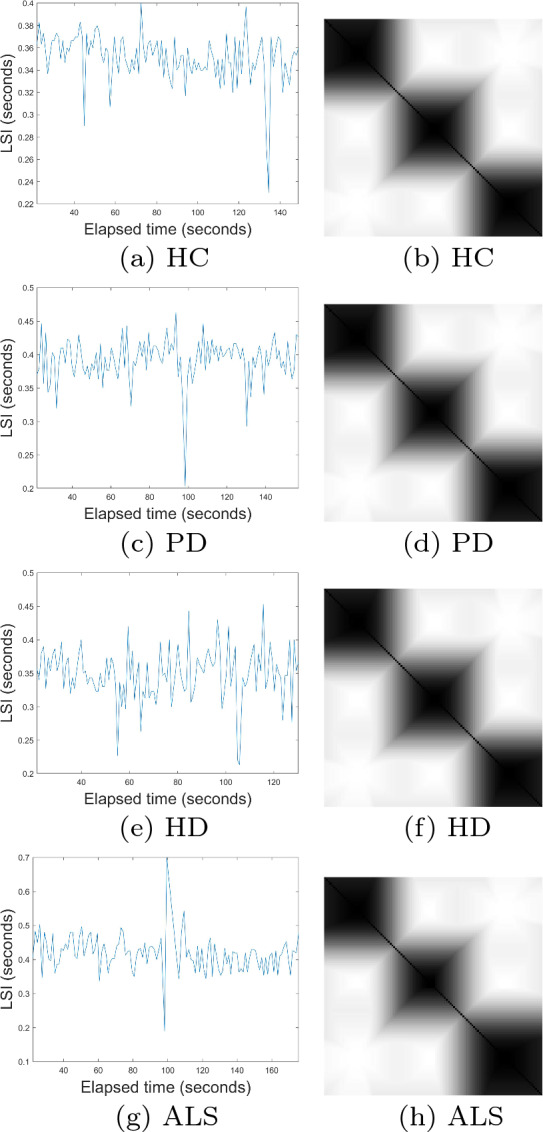
Time series of left swing intervals (left) and fuzzy recurrence plots (right) of healthy control and neurodegenerative subjects

**Fig. 4:**
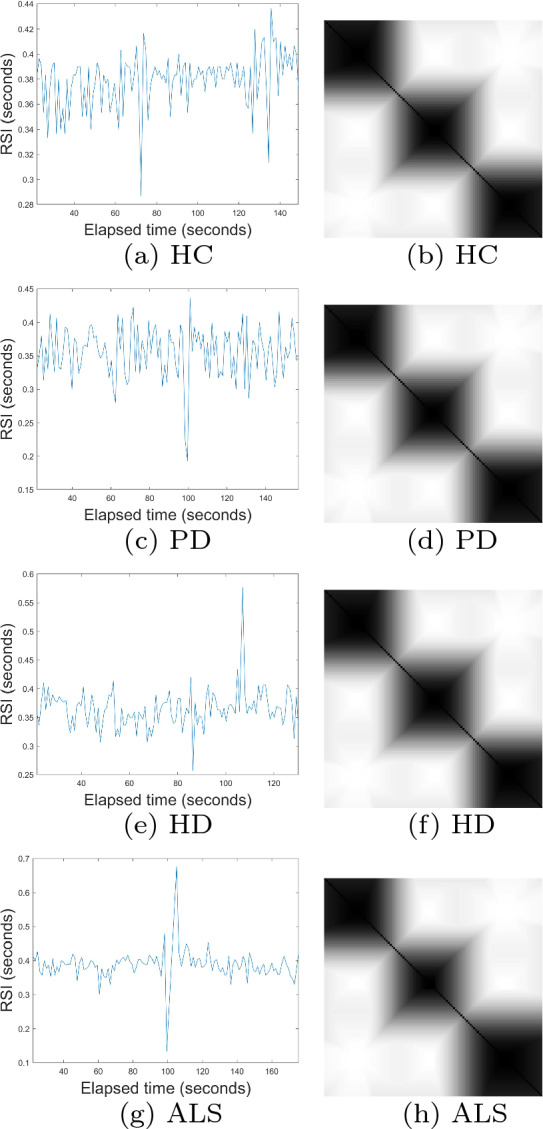
Time series of right swing intervals (left) and fuzzy recurrence plots (right) of healthy control and neurodegenerative subjects

To determine the largest eigenvalues from the time series of gait patterns of the HC, PD, HD, and ALS cohorts, the embedding dimension *m* = 1 was selected based on the one dimension of the signals, time delay $\tau$ = 1, and number of clusters *c* = 3. Figures 3 and 4 show some time series and FRPs of the HC, PD, HD, and ALS subjects. Being similar to the case of the *C. elegans*, to compute the largest eigenvalues, the size of the final cFRPs for the HC, PD, HD, and ALS time series was set as *n* = 2, that is a $2 \times 2$ matrix, to allow the deepest feature extraction based on the deep-learning approach.

Table 5 shows the average largest eigenvalues with standard deviations (SDs), *p*-values, 95% confidence intervals (95% CI), and 99% confidence intervals (99% CI) of the LSI and RSI of the HC, PD, HD, and ALS subjects. Table 6 shows the average SampEn values with standard deviations (SDs), *p*-values, 95% confidence intervals (95% CI), and 99% confidence intervals (99% CI) of the LSI and RSI of the HC, PD, HD, and ALS subjects. Input parameters for computing SampEn values of the time series were selected as: embedding dimension *m* = 2 (*m* = 1 resulting in some SampEn values = $\infty$), time delay $\tau$ = 1, and threshold $\delta$ = 0.3$\sigma$.

**Table 5:** Average largest eigenvalues and confidence intervals of left and right swing intervals

Cohort	Mean ± SD	*p*-value	95% CI	99% CI
Left swing interval				
HC	5.9875 ± 1.0132	2.7660e−13	(5.4476, 6.5274)	(5.2411, 6.7339)
PD	6.2174 ± 0.8175	5.3718e−14	(5.7647, 6.6701)	(5.5891, 6.8458)
HD	6.4782 ± 0.9651	1.7884e−17	(6.0266, 6.9299)	(5.8608, 7.0956)
ALS	6.3764 ± 1.0848	7.0810e−11	(5.7209, 7.0320)	(5.4574, 7.2954)
Right swing interval				
HC	6.3076 ± 1.1803	1.2009e−12	(5.6786, 6.9365)	(5.4380, 7.1771)
PD	6.5289 ± 1.2089	5.8534e−12	(5.8594, 7.1983)	(5.5997, 7.4581)
HD	6.5297 ± 0.9547	1.2600e−17	(6.0829, 6.9765)	(5.9190, 7.1404)
ALS	6.6049 ± 1.0707	4.0205e−11	(5.9579, 7.2519)	(5.6979, 7.5120)

**Table 6:** Average SampEn values and confidence intervals of left and right swing intervals

Cohort	Mean ± SD	*p*-value	95% CI	99% CI
Left swing interval				
HC	1.0265 ± 0.2934	5.1449e−10	(0.8702, 1.1829)	(0.8104, 1.2427)
PD	0.8603 ± 0.2849	1.2989e−08	(0.7025, 1.0180)	(0.6413, 1.0792)
HD	1.0914 ± 0.2077	1.6635e−15	(0.9942, 1.1886)	(0.9586, 1.2243)
ALS	0.9138 ± 0.2606	2.7036e−08	(0.7563, 1.0713)	(0.6930, 1.1346)
Right swing interval				
HC	0.9627 ± 0.3257	5.2881e−09	(0.7891, 1.1362)	(0.7228, 1.2026)
PD	0.8592 ± 0.3037	2.9780e−08	(0.6910, 1.0274)	(0.6258, 1.0927)
HD	1.0843 ± 0.2923	9.2497e−13	(0.9475, 1.2211)	(0.8973, 1.2713)
ALS	0.9523 ± 0.3480	4.1369e−07	(0.7420, 1.1625)	(0.6575, 1.2471)

## Discussion

The average largest eigenvalues of the eigenworm recurrence dynamics that were computed with the embedding dimension of 4 show consistently with evidence of statistical significance that the average largest eigenvalues of N2 (wild type/normal) are smaller than those of the four mutant worms with *c* = 3 and 5. The mutant type goa-63 has the average largest eigenvalues for *c* = 3 and 5. The goa-1 has the largest average largest eigenvalue for *c* = 7, but the average largest eigenvalue of N2 (5.2862) is slightly larger than unc-1 (5.2543). The mutant type unc-38 has the smallest average largest eigenvalues among the other three mutant types for both *c* = 3 and 5. However, the upper bounds of 95% and 99% CIs of the four mutants are higher than those of the wild type for all three different numbers of clusters. In general, these results suggest the use of *c* being either 3 or 5 for producing consistent results, and the use of the maximum eigenvalues of cFRPs can differentiate between wild type and mutant classes of *C. elegans*.

In contrast to the maximum eigenvalues, the average SampEn values of the wild type for all three thresholds are smaller than those of mutant types goa-1 and unc-1 but larger than those of mutant types unc-38 and unc-63. Such results suggest the SampEn method fails to differentiate between the wild type and mutant strains of *C. elegans*.

**Fig. 5:**
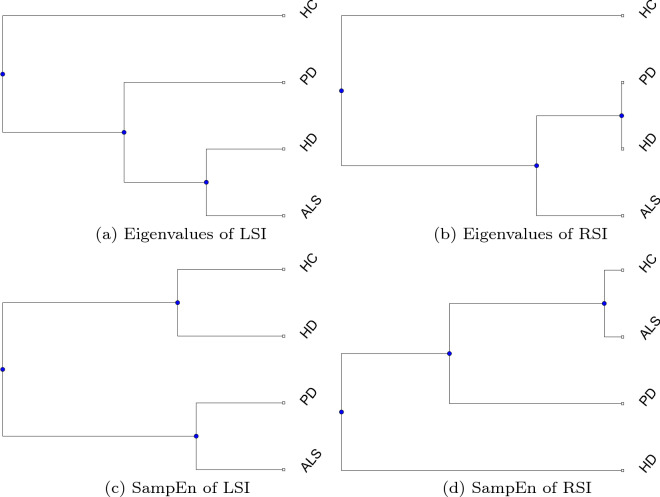
Phylogenetic trees constructed with UPGMA using pairwise distances of left and right swing intervals obtained from methods of recurrence eigenvalues and SampEn

For the analysis of gait dynamics, the results shown in Table 5 consistently indicate that the average eigenvalue of the HC is lower than those of the patients with neurodegenerative disorders using either LSI or RSI data. While the eigenvalues of PD, HD, and ALS are closer together, the eigenvalues of HD and ALS are closest for the case of LSI; but PD and HD are closest for RSI (see phylogenetic trees in Figure 5a, b, which were constructed using the unweighted pair group method with arithmetic mean (UPGMA) [38]). In both cases of LSI and RSI, the HC is clearly separated from the neurodegenerative−disorder cohort (Figure 5a, b).

On the contrary, values of the SampEn computed using either LSI and RSI time series as shown in Table 6 do not differentiate gait patterns between the HC and neurodegenerative-disorder subjects. These results can be visualized with the phylogenetic trees in Fig. 5c, d constructed using the UPGMA, where HC and HD are located in the same group (Fig. 5c) for the case of the LSI, and HC and ALS in the same group (Fig. 5d) for the RSI. Furthermore, for SampEn analysis of gait patterns of HC, PD, HD, and ALS subjects, it was found that for *m* = 1, SampEn = $\infty$ for some LSI and RSI time series. This was because no similarity between sub-segments of the time series had been detected, giving a conditional probability of zero and resulted in an infinite value of SampEn. Therefore, *m*=2 was then chosen.

Findings from the eigenvalues of gait suggest that LSI and RWI are potential markers of neurodegenerative disorders, which can help identify early stage of the diseases for optimal treatment and intervention.

In deep learning, which is the state-of-the-art of artificial intelligence, convolutional layers, which perform convolution operations, are the critical basic units implemented in convolutional neural networks. In functional analysis, a convolution is a mathematical operation on two functions or in this case it applies a kernel to an input, resulting in a transformed feature that expresses how the property of the original input is modified by the kernel. The iterative operation of the same kernel to the subsequent inputs results in a powerful feature map that can be used for classifying images of different objects. This application of the same kernel across an input image is a powerful idea [39].

In this study, the filter kernel was designed to sharpen edges or fuzzy information of the input FRP. The kernel, which was repeatedly applied across the FRP after the operations of the ReLU and max pooling, allowed the creation of deep features in the down-sampled FRPs. This ability of the AI method is commonly referred to as translational invariance that has been proven to be robust for addressing prediction problems [39]. This deep-learning mechanism offers an insight into the capability of the cFRP-based eigenvalues for identifying the dynamics of movements controlled by brain signals.

## Conclusion

The proposed algorithm for computing the largest eigenvalues of convolutional fuzzy recurrence plots of time series can differentiate normal from mutant types of *Caenorhabditis elegans*, and gait patterns between healthy control subjects and patients with neurodegenerative disorders. Methods for prediction of worm types by using time series of spatial movements recorded from the eigenworms are needed to assist life-science and neuroscience researchers to gain deeper understanding of the genetic basis of behavior and facilitate studies to probe how molecular, cellular, and systems-level approaches can use sensory inputs to infer neural circuits and behaviors [6] as well as other complex neural actions [40]. The eigenvalues of left and right swing intervals of gait dynamics can be useful for gaining insight into the dynamics of the sub-phases of the stride as well as effect of the diseases on gait asymmetry, and potentially used as markers of disease progresses.

The method for computing eigenvalues of a convolutional FRP appears to be a useful computational tool for nonlinear time-series analysis to quantify other types of behavioral dynamics and movements produced from brain signals. There are several other different filter kernels that can be explored and implemented either separately or parallelly to extract deep feature maps of FRPs to enhance the discriminatory power of the convolutional eigenvalue method for intraclass identification. Such a future study, if being successful, will be very useful because it can overcome the difficulty for using machine learning when training data are limited and the cost for data acquisition can be expensive.

## Data Availability

Publicly archived data supporting the results reported in the article can be found online: eigenworm data [23], and gait in neurodegenerative disease data [24]. MATLAB codes for reproducing the results are publicly available at the author’s personal homepage: https://sites.google.com/view/tuan-d-pham/codes under the names “Eigenvalues of eigenworm phenotypes” and “Eigenvalues of gait in neurodegenerative diseases”.
